# Entry of *Scotophilus* Bat Coronavirus-512 and Severe Acute Respiratory Syndrome Coronavirus in Human and Multiple Animal Cells

**DOI:** 10.3390/pathogens8040259

**Published:** 2019-11-22

**Authors:** Yi-Ning Chen, Hsiao-Chin Hsu, Sheng-Wei Wang, Hao-Chiang Lien, Hsin-Ti Lu, Sheng-Kai Peng

**Affiliations:** Department of Bioscience Technology, Chung Yuan Christian University, Taoyuan 32020, Taiwan; v4524369@gmail.com (H.-C.H.); steven_stevem@hotmail.com (S.-W.W.); muemily38@gmail.com (H.-C.L.); xj6vup2u4@gmail.com (H.-T.L.); s110028@shsh.tw (S.-K.P.)

**Keywords:** *Scotophilus* bat coronavirus-512, severe acute respiratory syndrome-coronavirus, pseudovirus, cell receptor

## Abstract

Bats are natural reservoirs of severe acute respiratory syndrome coronavirus (SARS-CoV) and Middle East respiratory syndrome CoV (MERS-CoV). *Scotophilus* bat CoV-512 demonstrates potential for cross-species transmission because its viral RNA and specific antibodies have been detected in three bat species of Taiwan. Understanding the cell tropism of *Scotophilus* bat CoV-512 is the first step for studying the mechanism of cross-species transmission. In this study, a lentivirus-based pseudovirus was produced using the spike (S) protein of *Scotophilus* bat CoV-512 or SARS-CoV as a surface protein to test the interaction between coronaviral S protein and its cell receptor on 11 different cells. Susceptible cells expressed red fluorescence protein (RFP) after the entry of RFP-bound green fluorescence protein (GFP)-fused S protein of *Scotophilus* bat CoV-512 (RFP-Sco-S-eGFP) or RFP-SARS-S pseudovirus, and firefly luciferase (FLuc) activity expressed by cells infected with FLuc-Sco-S-eGFP or FLuc-SARS-S pseudovirus was quantified. *Scotophilus* bat CoV-512 pseudovirus had significantly higher entry efficiencies in Madin Darby dog kidney epithelial cells (MDCK), black flying fox brain cells (Pabr), and rat small intestine epithelial cells (IEC-6). SARS-CoV pseudovirus had significantly higher entry efficiencies in human embryonic kidney epithelial cells (HEK-293T), pig kidney epithelial cells (PK15), and MDCK cells. These findings demonstrated that *Scotophilus* bat CoV-512 had a broad host range for cross-species transmission like SARS-CoV.

## 1. Introduction

Coronaviruses (CoVs) can cause severe diseases in humans and domestic animals. For instance, severe acute respiratory syndrome CoV (SARS-CoV) caused 8096 laboratory-confirmed cases with 774 deaths (10% mortality rate) between 2002 and 2003 [1], Middle East respiratory syndrome CoV (MERS-CoV) caused 2464 laboratory-confirmed cases with 850 deaths (34% mortality rate) from 2012 until 31 August in 2019 [2], porcine epidemic diarrhea virus (PEDV) and transmissible gastroenteritis virus (TGEV) caused high mortality in young pigs, infectious bronchitis virus (IBV) caused respiratory and renal diseases in chickens, and mouse hepatitis virus (MHV) caused hepatitis and demyelinating encephalitis in mice [3]. Since the outbreak of SARS-CoV, the highest number of novel CoV species has been discovered in many bat species [4]. Bat CoVs genetically close to SARS-CoV [5], MERS-CoV [6], human CoV (HCoV)-229E [7], and HCoV-NL63 [8] were detected and may contribute to the emergence of novel CoVs through several cross-species transmission events [4].

Bats are the natural reservoirs of SARS-CoV, MERS-CoV, and HCoV-NL63. SARS-CoV and HCoV-NL63 gain cellular entry through angiotensin-converting enzyme 2 (ACE2) [9], whereas MERS-CoV utilizes dipeptidyl peptidase-4 (DPP4) as its entry receptor [10]. The entry receptor is considered a hallmark of coronaviral cross-species transmissibility. SARS-related CoV isolated from *Rhinolophus* bats can use the ACE2 of humans, civets, and *Rhinolophus* bats as its cell receptor to infect cells originated from human and many other animal species [11]. Both MERS-CoV and bat CoV-HKU4 can use bat and human DPP4 to infect cells originating from humans, camels, bats, and other animal species [12,13] and HCoV-NL63 can replicate in the lung cell line from tricolored bats [8]. 

Little information is available regarding cross-species events for animal CoVs. A previous study detected *Scotophilus* bat CoV-512 in four different bat species along with *Miniopterus* bat CoV-1A and *Rhinolophus* SARS-related CoV in Taiwan through the reverse transcription polymerase chain reaction (RT-PCR) [14]. Antibodies specific to the nucleocapsid (N) protein fragments of *Scotophilus* bat CoV-512 were detected in serum collected from three bat species, namely *Scotophilus kuhlii*, *Miniopterus fuliginosus*, and *Rhinolophus monoceros* [15]. A close relationship and possible gene recombinants between *Scotophilus* bat CoV-512 and PEDV were observed through sequence alignments [14,16]. The results of the cell entry assay also indicated that PEDV can infect cells originating from pigs (PK15 and ST), humans (Huh-7 and MRC5), monkeys (Vero) and *Tadarida brasiliensis* bats (Tb1-Lu) [17]. We selected *Scotophilus* bat CoV-512 as our study target because it demonstrates the potential of cross-species infection and may play a role in the emergence or transmission of PEDV. Both molecular and serological evidences proved that *Scotophilus* bat CoV-512 can infect multiple animal species [14,15]. To investigate host tropisms contributing to cross-species transmission of *Scotophilus* bat CoV-512, we produced a pseudovirus bearing the full-length spike (S) protein of *Scotophilus* bat CoV-512 in the envelope (Env) protein-defective, red fluorescence protein (RFP)-expression or luciferase (FLuc)-expression human immunodeficiency virus type 1 (HIV-1) backbone for performing a cell entry assay in 11 different cells from humans, bats, dogs, cats, pigs, rats, and monkeys. 

## 2. Results

### 2.1. Generation of Primary Kidney Cells from Miniopterus fuliginosus 

Organs were prepared from individual bat and only primary kidney cells from one Eastern bent-winged bat (*Miniopterus fuliginosus*), named as MFK, were still viable after eight passages for performing a pseudovirus entry assay. Primary kidney cells from one Chestnut bat (*Scotophilus kuhlii*) and primary lung cells from one Formosa leaf-nosed bat (*Hipposideros armiger terasensis*) did not maintain their growth after three passages. 

### 2.2. Generation of Pseudoviruses

Lentivirus pseudotyped with the green fluorescence protein (GFP)-fused S protein of *Scotophilus* bat CoV-512 (Sco-S-eGFP), the S protein of SARS-CoV (SARS-S) or the glycoprotein (G) of vesicular stomatitis virus (VSV-G) was generated to study the cell entry of viruses in 11 different cells. The pseudovirus without the glycoprotein on the surface (△env) was produced as the control of mock infection. The generated pseudoviruses allowed for a single-cycle infection in different cells. After pseudoviruses carrying the transfer plasmid encoding red fluorescent protein (RFP) entered susceptible cells, RFP was expressed and observed under a microscope at 48 h post infection (hpi). Pseudoviruses carrying the transfer plasmid encoding luciferase (FLuc) were generated to quantify the entry efficiencies of pseudoviruses in susceptible cells by measuring luciferase activity at 48 hpi. 

#### 2.2.1. Pseudovirus Production after Co-Transfection 

**Figure 1:**
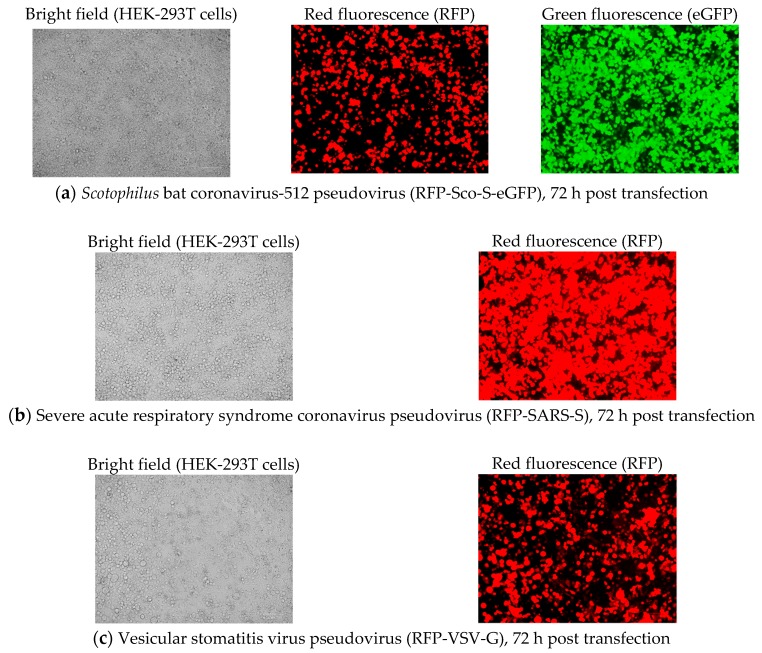
Pseudovirus production after co-transfection of three plasmids in human embryonic kidney (HEK)-293T cells at 72 h post transfection. (**a**) Production of red fluorescence protein (RFP)-bound green fluorescence protein (GFP)-fused S protein of *Scotophilus* bat coronavirus (CoV)-512 pseudovirus (RFP-Sco-S-eGFP): pictures from left to right are HEK-293T cells under bright field, red fluorescence produced by the expressed red fluorescence protein (RFP) from the transfer plasmid pLAS2w.RFP-C.Pneo, green fluorescence produced by the expressed green fluorescence protein (GFP) fused with the carboxyl terminal spike (S) protein of *Scotophilus* bat CoV-512. (**b**) Production of severe acute respiratory syndrome (SARS)-CoV (RFP-SARS-S): HEK-293T cells on the left and red fluorescence from RFP on the right. (**c**) Production of vesicular stomatitis virus (VSV) pseudovirus (RFP-VSV-G): HEK-293T cells on the left and red fluorescence from RFP on the right. The scale bars represent 100 µm.

To generate pseudotyped *Scotophilus* bat CoV-512 (RFP-Sco-S-eGFP), SARS-CoV (RFP-SARS-S), and VSV (RFP-VSV-G), three plasmids were co-transfected into human embryonic kidney epithelial cells (HEK-293T) cells. After 72 h post co-transfection with the packing plasmid (pCMVdeltaR8.91), transfer plasmid (pLAS2w.RFP-C.Pneo), and plasmid encoding the surface protein (pEGFP-Sco-S, pCMV-SARS-S, and pMD.G), red fluorescence from the expressed RFP was observed in HEK-293T cells producing the RFP-Sco-S-eGFP, RFP-SARS-S, or RFP-VSV-G pseudoviruses. Green fluorescence from the expressed GFP fused with the carboxyl terminal S protein of *Scotophilus* bat CoV-512 could be observed only in HEK-293T cells producing the RFP-Sco-S-eGFP pseudovirus (Figure 1). No fluorescence could be observed in HEK-293T cells producing the FLuc-SARS-S or FLuc-VSV-G pseudovirus carrying the transfer plasmid (pLAS2w-Fluc-Ppuro) encoding luciferase. Green fluorescence from the expressed GFP fused to the S protein of *Scotophilus* bat CoV-512 could be observed in HEK-293T cells producing the FLuc-Sco-S-eGFP pseudovirus. 

#### 2.2.2. Pseudovirus Titration 

**Figure 2:**
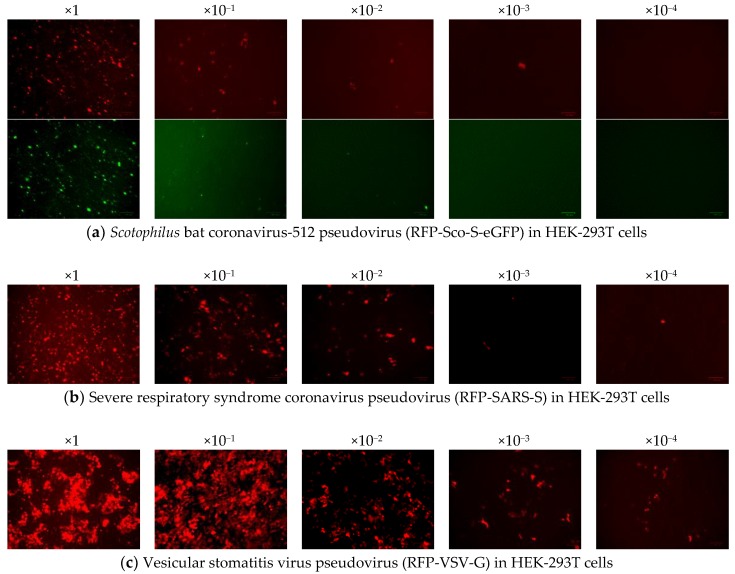
Pseudovirus titration from the dilutions of ×1 to ×10^−4^ into HEK-293T cells. (**a**) Infection of *Scotophilus* bat coronavirus-512 pseudovirus (RFP-Sco-S-eGFP) was indicated by the expression of red fluorescent protein (RFP) in cells on top panel and by the expression of green fluorescent protein (GFP) fused to the spike (S) protein on bottom panel. (**b**) Infection of severe acute respiratory syndrome coronavirus pseudovirus (RFP-SARS-S) was indicated by the expression of RFP. (**c**) Infection of vesicular stomatitis virus pseudovirus (RFP-VSV-G) was indicated by the expression of RFP. The scale bars represent 100 µm.

The infectivity of the generated pseudoviruses was determined by counting red fluorescent cells and also green fluorescent cells in the case of RFP-Sco-S-eGFP. The titers of the three pseudoviruses were calculated after the inoculation of serially tenfold-diluted pseudoviruses in HEK-293T cells (Figure 2). The average titers of RFP-Sco-S-eGFP, RFP-SARS-S, and RFP-VSV-G in HEK-293T cells were 5.4 × 10^4^, 2.4 × 10^4^, and 8 × 10^4^ TCID_50_/mL (infectious dose infecting 50% of tissue culture), respectively. For the pseudoviruses carrying the luciferase gene, the average titers of FLuc-Sco-S-eGFP, FLuc-SARS-S, and FLuc-VSV-G in HEK-293T cells were 3 × 10^2^, 1.5 × 10^3^, and 1.2 × 10^5^, respectively. The generated pseudoviruses were stored at −80 °C and virus titers began to decrease after 1 month of storage at −80 °C. Therefore, a fresh batch of pseudoviruses was prepared for pseudovirus entry assays performed for different batches of cells at different times.

#### 2.2.3. Detection of Pseudoviral Proteins after Infection

**Figure 3:**
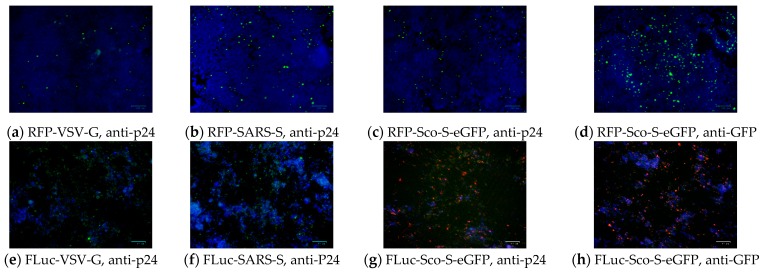
Detection of p24 protein in the lentiviral backbone of pseudoviruses and green fluorescence protein (GFP) fused with the spike protein of *Scotophilus* bat coronavirus-512 pseudovirus (RFP-Sco-S-eGFP) by immunofluorescence antibody (IFA) assay. (**a**) Positive green IFA signals for p24 in HEK-293T cells infected with vascular stomatitis virus pseudovirus (RFP-VSV-G). (**b**) Positive green IFA signals for p24 in HEK-293T cells infected with severe acute respiratory syndrome coronavirus pseudovirus (RFP-SARS-S). (**c**) Positive green IFA signals for p24 in HEK-293T cells infected with RFP-Sco-S-eGFP. (**d**) Positive green IFA signals for GFP in HEK-293T cells infected with RFP-Sco-S-eGFP. (**e**) Positive green IFA signals for p24 in MFK cells infected with FLuc-VSV-G. (**f**) Positive green IFA signals for p24 in MFK cells infected with FLuc-SARS-S. (**g**) Positive red IFA signals and green fluorescence from GFP in MFK cells infected with FLuc-Sco-S-eGFP. (**h**) Positive red IFA signals and green fluorescence from GFP in MFK cells infected with FLuc-Sco-S-eGFP. Blue parts are DAPI (4′,6-diamidino-2-phenylindole) staining cell nucleus. The scale bars represent 100 µm.

The detection of the p24 protein in the lentiviral backbone of pseudoviruses by using the immunofluorescent antibody assay (IFA) could confirm the infection of the six pseudoviruses. Cells infected with RFP-Sco-S-eGFP or FLuc-Sco-S-eGFP expressed not only RFP but also GFP, which could be observed directly or detected using the IFA. After 49 hpi, positive green IFA signals for p24 were observed in HEK-293T cells infected with RFP-Sco-S-eGFP, RFP-SARS-S, or RFP-VSV-G (Figure 3a–c). In addition, positive green IFA signals for GFP were observed in cells infected with RFP-Sco-S-eGFP (Figure 3d). After 48 hpi, positive green IFA signals for p24 were observed in MFK cells infected with FLuc-VSV-G or FLuc-SARS-S (Figure 3e,f). In cells infected with FLuc-Sco-S-eGFP, positive red IFA signals for GFP and green fluorescence produced by GFP could be observed and are shown in the merged images with red and green fluorescence images (Figure 3g,h).

#### 2.2.4. Electron Microscopic Morphology of Pseudoviruses 

**Figure 4:**
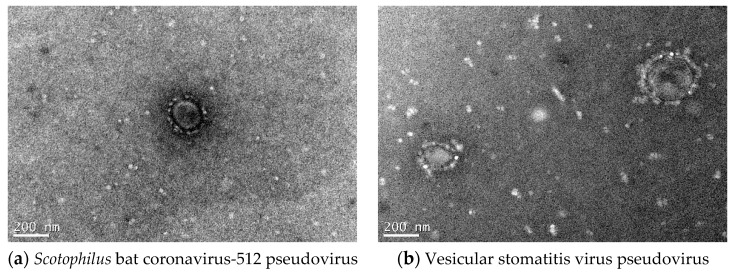
Electron microscopic images of pseudoviruses. (**a**) *Scotophilus* bat coronavirus-512 pseudovirus (RFP-Sco-S-eGFP). (**b**) Vesicular stomatitis virus pseudovirus (RFP-VSV-G).

The generated *Scotophilus* bat CoV-512 pseudoviruses RFP-Sco-S-eGFP showed a characteristic corona-structure and vesicular stomatitis virus pseudovirus RFP-VSV-G did not have corona-structure under the electron microscope (Figure 4). 

### 2.3. Cell Entries of RFP-Pseudoviruses

**Figure 5:**
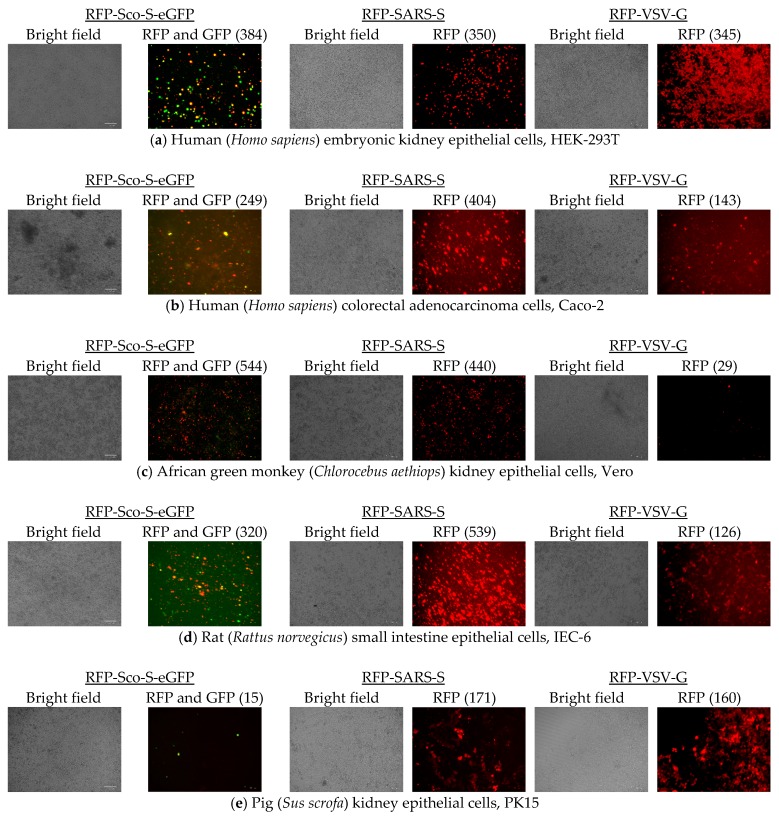

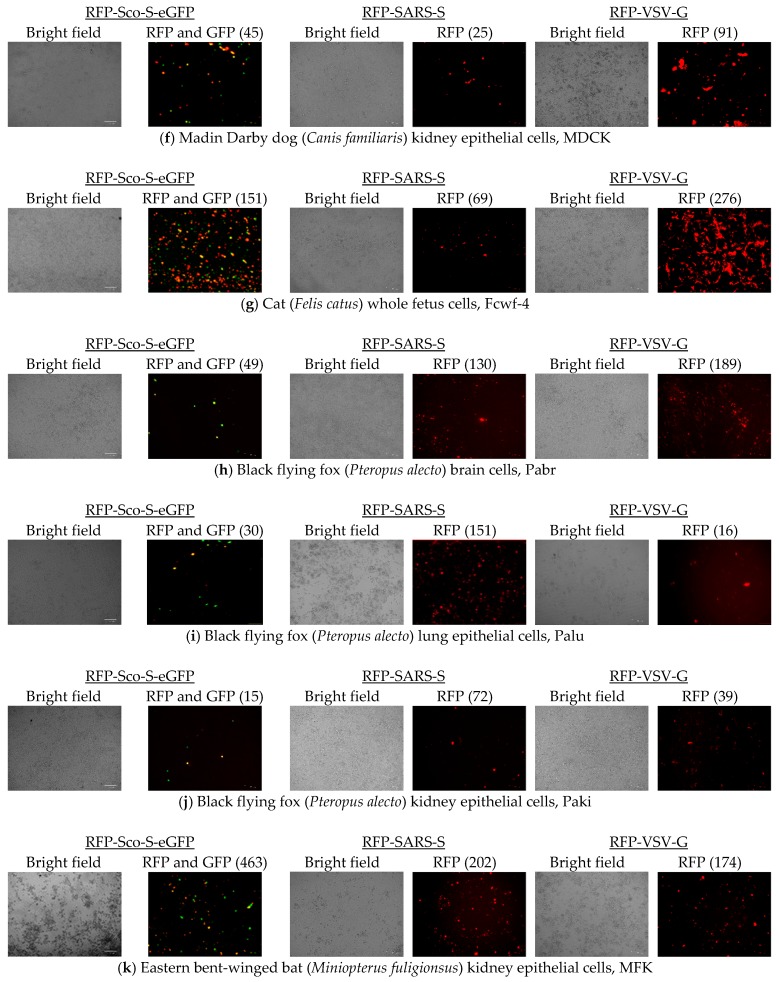
Cell entries of pseudoviruses carrying red fluorescence protein (RFP) gene are represented by red fluorescence images (RFP) for severe acute respiratory syndrome coronavirus pseudovirus (RFP-SARS-S, middle panels) and vesicular stomatitis virus pseudovirus (RFP-VSV-G, right panels), and merged red and green fluorescence images (RFP and GFP) present the entries of *Scotophilus* bat coronavirus-512 pseudovirus (RFP-Sco-S-eGFP, left panels) in 11 different cells. (**a**) HEK-293T: human (*Homo sapiens*) embryonic kidney epithelial cells; (**b**) Caco-2: human (*Homo sapiens*) colorectal adenocarcinoma cells; (**c**) Vero: African green monkey (*Chlorocebus aethiops*) kidney epithelial cells; (**d**) IEC-6: rat (*Rattus norvegicus*) small intestine epithelial cells; (**e**) PK15: pig (*Sus scrofa*) kidney epithelial cells; (**f**) MDCK: Madin Darby dog (*Canis* *familiaris*) kidney epithelial cells; (**g**) Fcwf-4: cat (*Felis catus*) whole fetus cells; (**h**) Pabr: black flying fox (Pteropus alecto) brain cells; (**i**) Palu: black flying fox (*Pteropus alecto*) lung epithelial cells; (**j**) Paki: black flying fox (*Pteropus alecto*) kidney epithelial cells; (**k**) MFK: Eastern bent-winged bat (*Miniopterus fuliginosus*) kidney epithelial cells. Bright field images of cells were used as reference. The scale bars represent 100 µm.

Cell entries of RFP-Sco-S-eGFP, RFP-SARS-S, and RFP-VSV-G were determined by counting red fluorescent cells and all tested cells were susceptible to the infection of the three pseudoviruses (Figure 5). At 48 hpi of the pseudovirus RFP-Sco-S-eGFP, more than 100 cells showing red fluorescence per field were observed in Vero (average 544 red fluorescent cells), MFK (463), 293T (384), IEC-6 (320), Caco-2 (249), and Fcwf-4 (151) cells and less than 50 cells showing red fluorescence per field were observed in Pabr (49), MDCK (45), Palu (30), Paki (15), and PK15 (15) cells. Cells infected with RFP-Sco-S-eGFP also showed green fluorescence from the expressed GFP fused to the S protein of *Scotophilus* bat CoV-512. At 48 hpi of the pseudovirus RFP-SARS-S, more than 100 cells showing red fluorescence per field were observed in IEC-6 (539), Vero (440), Caco-2 (404), 293T (350), MFK (202), PK15 (171), Palu (151), and Pabr (130) cells and less than 80 cells showing red fluorescence per field were observed in Paki (72), Fcwf-4 (69), and MDCK (25) cells. At 48 hpi of the pseudovirus RFP-VSV-G, more than 100 cells showing red fluorescence per field were observed in 293T (345), Fcwf-4 (276), Pabr (189), MFK (174), PK15 (160), Caco-2 (143), and IEC-6 (126) cells and less 100 cells showing red fluorescence per field were observed in MDCK (91), Paki (39), Vero (29), and Palu (16) cells. 

### 2.4. Cell Entries of FLuc-Pseudoviruses

**Figure 6:**
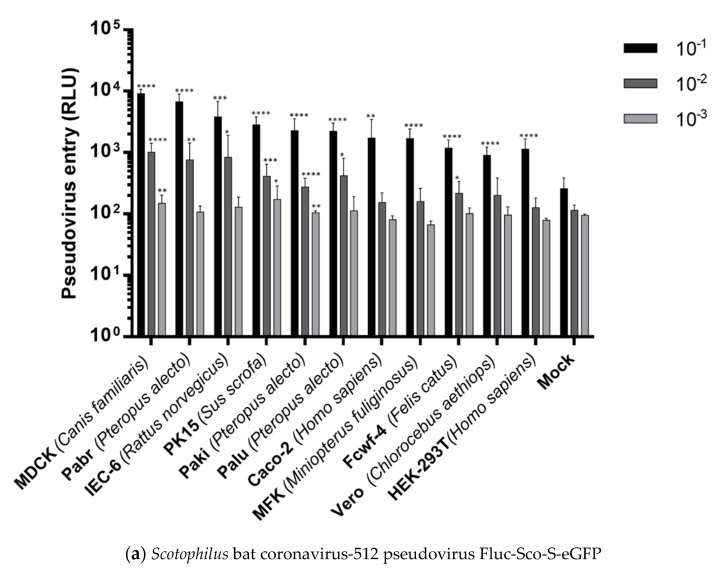

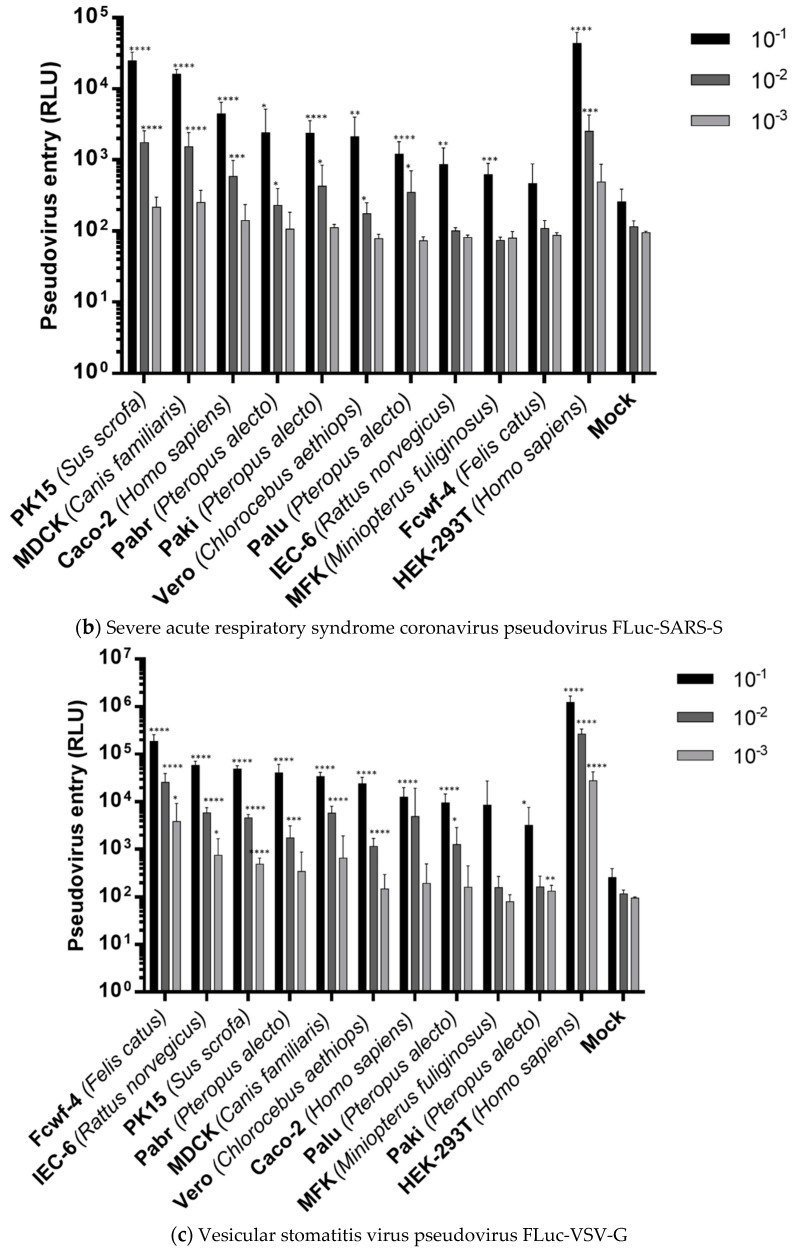
Cell entry efficiencies of pseudoviruses diluted in 10^−1^, 10^−2^, and 10^−3^ were measured by luciferase activity (RLU) at 48 h post infection. (**a**) Infection by *Scotophilus* bat coronavirus-512 pseudovirus (FLuc-Sco-S-eGFP). (**b**) Infection by severe acute respiratory syndrome coronavirus pseudovirus (FLuc-SARS-S). (**c**) Infection by vesicular stomatitis virus pseudovirus (FLuc-VSV-G). Mock presents the HEK-293T cells inoculated with the pseudovirus without surface glycoprotein (△env) as negative control. HEK-293T: human (*Homo sapiens*) embryonic kidney epithelial cells; Caco-2: human (*Homo sapiens*) colorectal adenocarcinoma cells; Vero: African green monkey (*Chlorocebus aethiops*) kidney epithelial cells; IEC-6: rat (*Rattus norvegicus*) small intestine epithelial cells; PK15: pig (*Sus scrofa*) kidney epithelial cells; MDCK: Madin Darby dog (*Canis familiaris*) kidney epithelial cells; Fcwf-4: cat (*Felis catus*) whole fetus cells; Pabr: black flying fox (*Pteropus alecto*) brain cells; Palu: black flying fox (*Pteropus alecto*) lung epithelial cells; Paki: black flying fox (*Pteropus alecto*) kidney epithelial cells; MFK: Eastern bent-winged bat (*Miniopterus fuliginosus*) kidney epithelial cells. Error bars indicate the standard deviation (n = 12). RLU values from each set of cells were compared to RLU value of Mock infection in 293T cells by using multiple *t* tests (* *p* < 0.05; ** *p* < 0.01; *** *p* < 001; **** *p* < 0.0001).

After the inoculation of tenfold-diluted (10^−1^) pseudovirus FLuc-Sco-S-eGFP, FLuc-SARS-S, or FLuc-VSV-G, all 11 tested cells showed luciferase activity (RLU) stronger than those from 293T cells inoculated with the pseudovirus without the glycoprotein on the surface (△env) as mock infection control (Figure 6). Dilution effects were observed between the cells inoculated with 10^−1^, 10^−2^, or 10^−3^ of pseudoviruses. At 48 hpi of FLuc-Sco-S-eGFP, MDCK had average RLU value of 9126, which was the highest RLU among 11 cells followed by Pabr (6708), IEC-6 (3822), PK15 (2835), Paki (2270), Palu (2225), Caco-2 (1715), 293T (2804), MFK (1683), Fcwf-4 (1188), and Vero (909) cells. At 48 hpi of FLuc-SARS-S diluted in 10^−1^, 293T (25,136) cells had the highest RLU followed by PK15 (24,723), MDCK (16,024), Caco-2 (4463), Pabr (2398), Paki (2382), Vero (2118), Palu (1201), IEC-6 (859), MFK (623), and Fcwf-4 (466) cells. At 48 hpi of FLuc-VSV-G diluted in 10^−1^, 293T (815,628) cells had the highest RLU followed by Fcwf-4 (185,952), IEC-6 (57,767), PK15 (48,325), Pabr (40,356), MDCK (33,626), Vero (20,886), Caco-2 (12,466), Palu (9510), MFK (8477), and Paki (3181) cells. All 11 cells were susceptible to *Scotophilus* bat CoV-512 because the RLU vales of all 11 cells inoculated with 10^−1^ of pseudovirus were significantly higher than those of mock infection (*p* < 0.01 to *p* < 0.0001). All tested cells except Fcwf-4 cells showed significantly higher RLU values than those of mock infection and they were susceptible to SARS-CoV (*p* < 0.05 to *p* < 0.0001). For VSV, all tested cells except MFK cells showed significantly higher RLU values than those of mock infection (*p* < 0.05 to *p* < 0.0001). Some cells could still produce significantly higher RLU values than those from mock infection after the inoculation of pseudovirus diluted into 10^−2^ and 10^−3^ (*p* < 0.05 to *p* < 0.0001).

Supplementary Figure S1 shows the entry efficiencies of different pseudoviruses in tenth dilution (10^−1^) on the basis of cell lines to allow a more direct comparison of virus infectivity in one species. Pseudovirus FLuc-VSV-G had the highest entry efficiencies that other two pseudoviruses did in all cells except Palu, where FLuc-Sco-S-eGFP had the highest entry efficiency. No significant differences were observed in Vero, Paki, and MDCK cells infected with FLuc-Sco-S-eGFP and FLuc-SARS-S pseudoviruses, respectively. Pseudovirus FLuc-Sco-S-eGFP had significantly higher entry efficiencies than FLuc-SARS-S did in IEC-6, Pabr, Palu, and Fcwf-4 cells. From an evolutionary perspective, FLuc-Sco-S-eGFP had higher entry efficiencies in bat cells than FLuc-SARS-S did even though FLuc-SARS-S had significantly higher entry efficiency in MFK cells than FLuc-Sco-S-eGFP did.

### 2.5. Detection of Cell Receptors on Different Cells 

**Figure 7:**
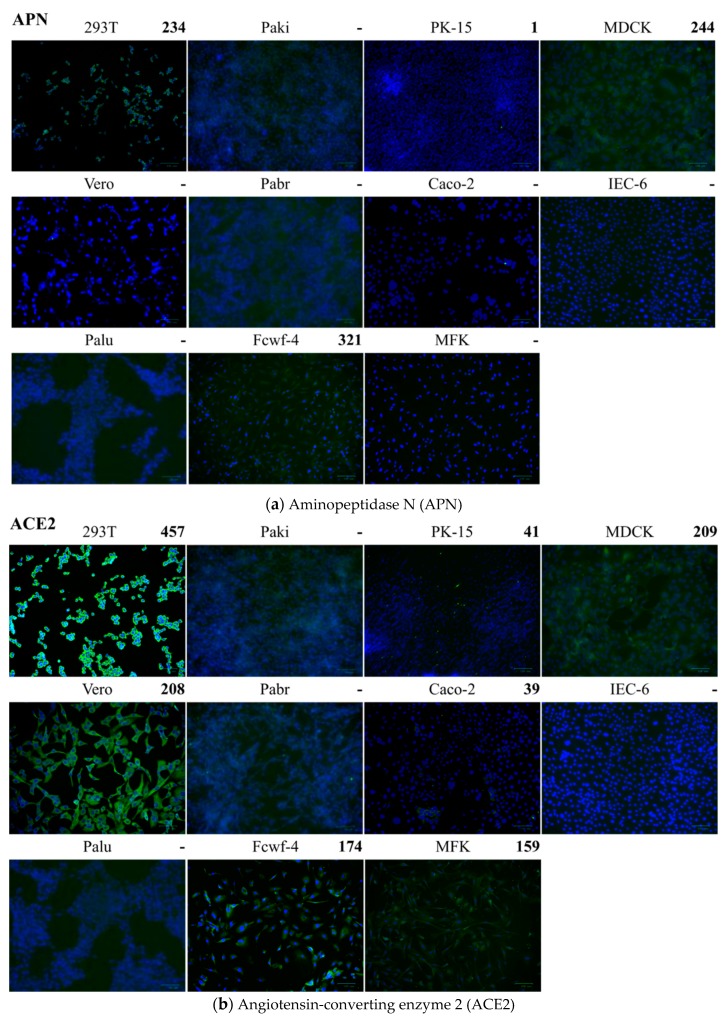

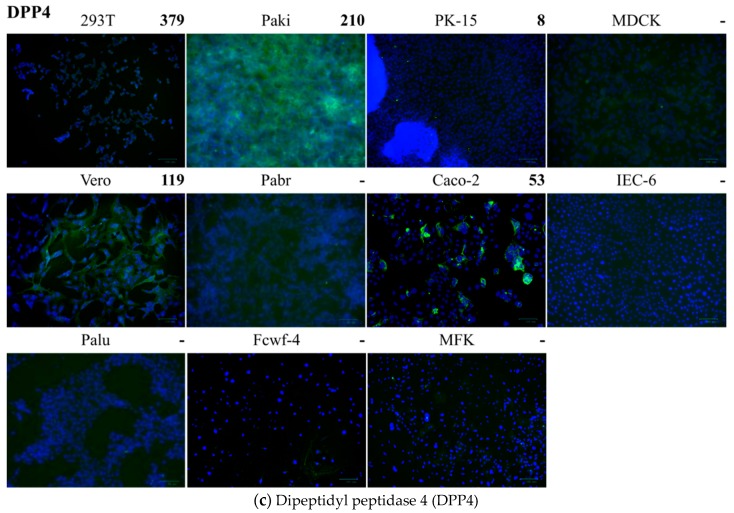
Detection of cell receptors by immunofluorescence antibody (IFA) assay. (**a**) Aminopeptidase N (APN) detected by 1:100 rabbit anti-human APN antibody (Bioss). (**b**) Angiotensin-converting enzyme 2 (ACE2) detected by 1:200 rabbit anti-human ACE2 antibody (abcam). (**c**) Dipeptidyl peptidase-4 (DPP4) detected by 1:00 goat anti-human DPP4 antibody (R&D). Green fluorescent signals indicated IFA positive responses. Blue parts were DAPI staining cell nucleus. 293T: human (*Homo sapiens*) embryonic kidney epithelial cells; Caco-2: human (*Homo sapiens*) colorectal adenocarcinoma cells; Vero: African green monkey (*Chlorocebus aethiops*) kidney epithelial cells; IEC-6: rat (*Rattus norvegicus*) small intestine epithelial cells; PK15: pig (*Sus scrofa*) kidney epithelial cells; MDCK: Madin Darby dog (*Canis familiaris*) kidney epithelial cells; Fcwf-4: cat (*Felis catus*) whole fetus cells; Pabr: black flying fox (*Pteropus alecto*) brain cells; Palu: black flying fox (*Pteropus alecto*) lung epithelial cells; Paki: black flying fox (*Pteropus alecto*) kidney epithelial cells; MFK: Eastern bent-winged bat (*Miniopterus fuliginosus*) kidney epithelial cells. The scale bars represent 100 µm.

The number of cells reacted to the antibodies specific to aminopeptidase N (APN), ACE2, or DPP4 was counted by Image J program (Figure 7). Strong positive IFA responses meant there were more than 200 positive cells per microscopic field and weak positive IFA responses meant less than 200 positive cells per microscopic field. HEK-293T, MDCK, and Fcwf-4 cells had strong positive IFA responses and PK15 had a weak positive IFA response to APN. HEK-293T, MDCK, and Vero cells had strong positive IFA responses and PK15, Caco-2, Fcwf-4, and MFK cells had weak positive IFA responses to ACE2. HEK-293T and Paki cells had strong positive IFA responses and PK15, Vero, and Caco-2 cells had weak positive IFA responses to DPP4. In summary, HEK-293T and PK15 cells were tested positive for APN, ACE2, and DPP4. Vero and Caco-2 cells were tested positive for ACE2 and DPP4. Fcwf-4 and MDCK cells were tested positive for APN and ACE2. MFK cells were tested positive only for ACE2. Paki cells were tested positive only for DPP4. Pabr, ICE-6, and Palu cells were tested negative for APN, ACE2, and DPP4. The results of cell receptors detection (Figure 7) and FLuc pseudovirus entry assay (Figure 6 and Supplementary Figure S1) are summarized in Table 1.

## 3. Discussion

In this study, entry efficiencies of *Scotophilus* bat CoV-512 and SARS-CoV in 11 different cells were evaluated using a lentivirus-based pseudovirus system. HEK-293T cells were selected as they have been used for the production of pseudoviruses; additionally, several cell lines were selected because they were used for the isolation and maintenance of viruses in other studies, such as Vero and PK15 cells for PEDV [17], MDCK cells for influenza viruses [18], and Fcwf-4 cells for FCoV [19]. Given that CoVs tend to replicate in the epithelial cells of enteric and respiratory tracts, Caco-2 and IEC-6 were selected. MFK cells were selected because viral RNA and specific antibodies of *Scotophilus* bat CoV-512 were detected in *Miniopterus fuliginosus*. *Pteropus* bat-cell lines (Paki, Pabr, Palu) were selected as alternative bat cells for comparison. All the 11 tested cells were susceptible to the entry of *Scotophilus* bat CoV-512 or SARS-CoV pseudovirus in varying degrees. *Scotophilus* bat CoV-512 pseudovirus had significantly higher entry efficiencies in MDCK, Pabr, and IEC-6 cells, followed by PK15, HEK-293T, Paki, Palu, Caco-2, MFK, Fcwf-4, and Vero cells. Notably, only MDCK, PK15, HEK-293T, and Fcwf-4 cells tested positive for APN, the proposed cell receptor for PEDV [20]. However, APN as a cell receptor of PEDV is not completely conclusive, and conflicting results have demonstrated blockage of PEDV infection by pAPN antibodies [20], although soluble APN could not inhibit PEDV infection [21]. As porcine APN-knockout swine testis cells did not inhibit PEDV infection [21] and APN-knockout pigs are not resistant to PEDV infection [22], APN may not be required for PEDV cell entry but it can promote the infectivity through aminopeptidase activity [23]. In this study, polybrene, trypsin or other proteases were not used for the pseudovirus entry assay; therefore, the cell entry of pseudovirus was determined by receptor recognition, implying that *Scotophilus* bat CoV-512 could enter susceptible cells via cell receptors other than APN.

**Figure 8:**
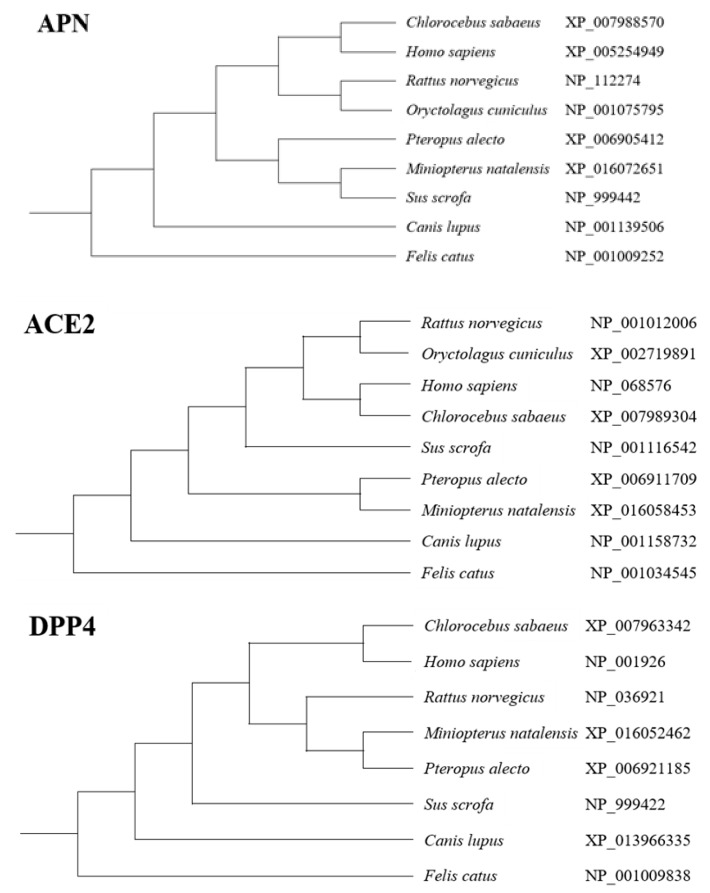
Phylogenetic trees of aminopeptidase N (APN), angiotensin-converting enzyme-2 (ACE2) and dipeptidyl peptidase-4 (DPP4) from the animal species tested in this study. HEK-293T: human (*Homo sapiens*) embryonic kidney epithelial cells; Caco-2: human (*Homo sapiens*) colorectal adenocarcinoma cells; Vero: African green monkey (*Chlorocebus aethiops*) kidney epithelial cells; IEC-6: rat (*Rattus norvegicus*) small intestine epithelial cells; PK15: pig (*Sus scrofa*) kidney epithelial cells; MDCK: Madin Darby dog (*Canis familiaris*) kidney epithelial cells; Fcwf-4: cat (*Felis catus*) whole fetus cells; Pabr: black flying fox (*Pteropus alecto*) brain cells; Palu: black flying fox (*Pteropus alecto*) lung epithelial cells; Paki: black flying fox (*Pteropus alecto*) kidney epithelial cells; MFK: Eastern bent-winged bat (*Miniopterus fuliginosus*) kidney epithelial cells.

The polyclonal antibody to human APN (Bioss) used in this study was produced in the rabbits inoculated with a keyhole limpet hemocyanin (KLH)-conjugated synthetic peptide derived from human APN. Its cross-reactivity was tested by Bioss company and it can cross-react to mouse and rat APN. We compared the sequences of APN from the animal species of the tested cells in this study (Figure 8), the APN of Eastern bent-winged bat (*Miniopterus fuliginosus*) had the closest phylogenetic relationship with pig (*Sus scrofa*) APN, followed by black flying fox (*Pteropus alecto*) APN. Interestingly, human APN had closer phylogenetic relationship with bat APN than dog and cat APN. Therefore, the negative IFA results to anti-APN antibody in Pabr cells from black flying fox and MFK cells from Eastern bent-winged bat could result from the lack of APN receptor but not from the lack of cross-reactivity of anti-APN antibody because MDCK cells from dogs and Fcwf-4 cells from cats showed strong positive IFA signals. There was no correlation between the phylogenetic relationship of APNs and the infectivity pattern of *Scotophilus* bat CoV-512. For ACE2, human ACE2 had the closest relationship to monkey ACE2, followed by rat, pig, bats, dogs, and cats (Figure 8). The polyclonal antibody to human ACE2 provided by abcam company has cross-reactivity to ACE2 from cat, ferret, and macaque monkey. Interestingly, the phylogenetic tree of ACE2 could roughly match the infectivity pattern of SARS-CoV. The findings indicate that *Scotophilus* bat CoV-512 may have different evolutionary perspective on cross-species transmission than SARS-CoV. In future study, we will try to identify the cell receptor used by *Scotophilus* bat CoV-512 and use real-time PCR with species-specific primers to quantify the mRNA levels of the identified cell receptor because the detection of cell receptors by using IFA would encounter the limitations of antibodies unable to bind to the cell receptors of different animal species.

APN was identified as the cell receptor of HCoV-229E, TGEV, FCoV, and possibly PEDV. In human, APN is mostly expressed in epithelial cells of the kidney, intestine, and respiratory tract; granulocytes, monocytes, fibroblasts, endothelial cells, cerebral pericytes at the blood–brain barrier, synaptic membranes of cells in the central nerve system (CNS). The highest expression levels of APN can be found in the small intestinal and renal tubular epithelial cells [24]. The tissue specificity and distribution of APN were correlated with the high entry efficiencies of FLuc-Sco-S-eGFP pseudovirus in dog kidney cells (MDCK), black flying fox brain cells (Pabr), rat small intestine cells (IEC-6), pig kidney cells (PK15), and black flying fox kidney cells (Paki). However, different animal species may have different tissue specificity and distribution of APN even though little information is available. According to the studies on DPP4 distribution, insectivorous bats primarily express DPP4 in the gastro-intestinal (GI) tract and kidneys, frugivorous bats express DPP4 in the respiratory and GI tracts [25], camels express DPP4 in the upper respiratory tract [26], and humans express DPP4 mainly in the lower respiratory tract [27]. Different tissue distribution of DPP4 in different animal species leads to different tissue tropism of MERS-CoV and varied disease severity [24,25,26,27]. ACE2 was identified as the cell receptor of SARS-CoV and HCoV-NL63 although two CoVs bind to different parts of ACE2 [28]. The highest expression levels of ACE2 are found in small intestine, duodenum, gall bladder, kidney, testis, and heart [24]. All three cells showing the highest entry efficiencies of FLuc-SARS-S pseudovirus were kidney epithelial cells: HEK-293T, PK15, and MDCK cells, and they all tested positive for ACE2. 

CoV spike proteins require proteolytic activation to mediate virus entry into cells but different CoVs use different types of proteases for their cell entry mechanism. PEDV uses lysosomal cysteine proteases (cathepsin L and cathepsin B) to activate S protein, whereas other CoVs use proprotein convertases or cell-surface serine proteases [29]. Extracellular trypsin could activate the entry of PEDV, SARS-CoV and MERS-CoV into host cells [29]. *Scotophilus* bat CoV-512 is more closely related to PEDV than to other known alpha-CoVs, such as TGEV, HCoV-229E, and HCoV-NL63 [14,16,30]. Although *Scotophilus* bat CoV-512 may have an entry mechanism similar to that of PEDV, differences are expected because amino acid sequence identity between the S proteins from *Scotophilus* bat CoV-512 and PEDV is only 48% for the S1 subunit containing the receptor-binding domain [17]. For example, although MDCK was not susceptible to PEDV [17], it showed the highest luciferase activity for the entry of *Scotophilus* bat CoV-512. Both *Scotophilus* bat CoV-512 and PEDV can enter PK15 [17] due to the potential gene recombination between the two CoVs during co-infection. Further studies are warranted to verify the types of requisite proteases for proteolytic activation of the S protein for cell entry by *Scotophilus* bat CoV-512.

SARS-CoV pseudovirus had significantly higher entry efficiencies in HEK-293, PK15, and MDCK cells, followed by Caco-2, Pabr, Paki, Vero, Palu, IEC-6, MFK, and Fcwf-4 cells. Studies have shown Vero, Caco-2, PK15, and HEK-293T cells as susceptible to infection by live SARS-CoV Urbani and HKU 39849 strains, but not MDCK cells even though these cells can express ACE2 [31,32]. HEK-293T, Vero, and Caco-2 cells were also susceptible to the entry of SARS-CoV pseudovirus; however, no data about the susceptibility of MDCK cells to SARS-CoV pseudovirus are available [33]. Strong luciferase activity detected in the MDCK cells inoculated with FLuc-SARS-S pseudovirus indicated viral entry mediated by the binding between SARS-CoV S protein and ACE2 on the surface of MDCK cells without interference from other cellular factors, such as glycosylation on S protein [34] or ACE2 [35], which can cause MDCK cells to resist the infection by live SARS-CoV [31]. Moreover, different glycosylation profiles can determine the host range of CoVs. For example, human APN can lose or gain its ability to support HCoV-229E infection when a glycosylation site is added to human APN or removed from the mouse APN, respectively [36,37]. Additionally, the addition of a glycosylation site to the rat ACE2 allows it to support SARS-CoV infection [38]. Further studies on glycosylation profiles can be insightful on the range of hosts of *Scotophilus* bat CoV-512 once its cell receptor is identified.

Coronavirus infection and replication are very complex processes requiring many viral and host proteins. We used the pseudoviruses carrying S protein of *Scotophilus* bat CoV-512 or SARS-CoV to study the cell entry of CoV. There are still many questions to be answered after we identified the cells susceptible to *Scotophilus* bat CoV-512 or SARS-CoV, including the identification of cell receptor for *Scotophilus* bat CoV-512, the glycosylation patterns of S protein and cell receptor, and the usage of viral and host proteases. In addition to cell entry, successful cross-species transmission requires viral replication and strategies to evade the innate immune responses of the new hosts. Marburg virus study showed that the increase in virus replication in bats may correlate with an increased incidence of human spillover events [39]. The correlations between virus replication rates and cross-species transmission of CoVs have not been explored for bats. Replicase and E protein are essential for CoV replication [40,41]. Accessory proteins of CoVs are not required for virus replication in cultured cells [42] but may play an important role in pathogenesis in natural hosts [43]. Viral proteins, in particular nsp1 [44], nsp3 [45], N protein and the SARS-CoV accessary protein ORF6, ORF3b [46], and ORF4a [47], prevent IFN induction. *Scotophilus* bat CoV-512 would need to evolve a strategy to overcome the innate immune responses in new host cells. 

The susceptibility of bat cells to MERS-CoV has been increasingly studied since the first laboratory-confirmed case in 2012 because of its close phylogenetic relationships with *Tylonycteris* bat CoV-HKU4, *Pipistrellus* bat CoV-HKU5 [6], and *Hypsugo pulveratus* bat CoV-HKU25 [48]. Twelve primary bat-cell lines from seven bat species were tested for their susceptibility to MERS-CoV, SARS-CoV, and HCoV-229E [49]. While HCoV-229E cannot infect any bat cells and SARS-CoV can only infect kidney cells of *Rhinolophus sinicus*, MERS-CoV can infect five different bat cells from four different bat species; additionally, Muller’s study demonstrated that MERS-CoV could infect five different bat cells from five different bat species [49,50]. These findings indicate that MERS-CoV has a much broader cell tropism than SARS-CoV and HCoV-229E. Although, in this study, MERS-CoV pseudovirus was not tested, IFA was used to determine the distribution of DPP4 on the 11 tested cells. A previous study showed that DPP4 surface expression varied between species but did not correlate with infectivity [51] although significantly higher mRNA expression levels of DPP4 were detected in the five bat cells susceptible to MERS-CoV than those in non-susceptible cells [49]. In the future, comparison of susceptibility of these cells to *Scotophilus* bat CoV-512 and MERS-CoV should be noteworthy. 

## 4. Materials and Methods 

### 4.1. Animal Experiment Ethics

The protocol for capturing and sampling of bats was approved by the Chung Yuan Christian University Animal Care and Use committee and the agriculture bureau of regional government (No. 105024). 

### 4.2. Primary Bat Cell Culture

One Eastern bent-winged bat (*Miniopterus fuliginosus*) was captured for collecting samples for CoV detection and euthanized later due to a complex bone fracture of the left wing. Lung, liver, kidney, heart, spleen, and intestine were collected and diced into small pieces. After the tissue pieces were washed with cold processing medium (Mg^2+^ and Ca^2+^-free phosphate-buffered saline (PBS) containing 200 mg/L disodium EDTA, 100 unit/mL penicillin, 100 µg/mL streptomycin), cold 0.25% trypsin in PBS containing 200 mg/L disodium ethylenediaminetetraacetic acid (EDTA) was added for the incubation at 4 °C overnight. Next, the tubes containing tissue were incubated at 37 °C on a shaking platform for 1 h. Trypsin-treated cells were pelleted by centrifugation at 800× g for 5 min and then re-suspended into Gibco™ Eagle’s Minimal Essential Medium containing Earle’s salts (EMEM, Thermo Fisher, Waltham, MA, USA) containing 1× penicillin/streptomycin and 10% fetal bovine serum (FBS, Biological Industries, Cromwell, CT, USA)After 3 to 5 days of incubation in a humidified incubator with 5% CO_2_ at 37 °C, the cells were passaged and maintained up to eight passages. Cell culture medium for propagation was DMEM with 15% FBS, 1.5 g NaHCO_3_, 1 mM sodium pyruvate, and 1× penicillin–streptomycin–amphotericin B solution. The primary kidney cells were named as *Miniopterus fuliginous* kidney cells, MFK.

### 4.3. Cell Line Culture

Ten cell lines from different organs and animal species were chosen in this study (Table 1). Cell culture medium for HEK-293T, MDCK, Paki, Pabr, Palu, and PK15 cells was Gibco™ Dulbecco’s Modified Eagle Medium (DMEM) (Thermo Fisher) with 10% FBS (Biological Industries), 1.5 g NaHCO_3_, 1 mM sodium pyruvate (Thermo Fisher), and 1× penicillin–streptomycin–amphotericin B solution (Biological Industries). Cell culture medium for Vero, Caco-2, and Fcwf-4 cells was Minimum Essential Medium Eagle (MEM) (Sigma-Aldrich, St. Louis, MO, USA) with 10% FBS, 1.5 g NaHCO_3_, 1 mM sodium pyruvate, 0.01% NEAA (Thermo Fisher), and 1× penicillin–streptomycin–amphotericin B solution. Cell culture medium for IEC-6 was DMEM with 5% FBS, 0.1 U/mL 95% bovine insulin (Sigma-Aldrich), 1.5g NaHCO_3_, 1 mM sodium pyruvate, and 1× penicillin–streptomycin–amphotericin B solution. Eleven cells were divided into four groups according to their growth rate. HEK-293T, Paki, PK15, and MDCK cells belonged to the first group and grew to 70% confluence for one day. Vero, Pabr, Caco-2, and IEC-6 cells belonged to the second group and grew to 70% confluence for two days. Palu and Fcwf-4 cells belonged to the third group and grew to 70% confluence for three days. MFK cells needed four days to grow to 70% confluence and belonged to the fourth group. 

### 4.4. Pseudovirus Generation

Non-replicative lentivirus system from the RNAi core at Academia Sinica was used to generate two types of pseudovirus: one carried the reporter gene encoding red fluorescence protein (RFP) and another one carried the reporter gene encoding firefly luciferase (FLuc). HEK-293T cells were co-transfected with packaging plasmid pCMVdeltaR8.91, transfer plasmid pLAS2w.RFP-C.Pneo or pLAS2w.FLuc.Ppuro, and surface protein plasmid containing the designed gene encoding surface protein (pEGFP-Sco-S for Sco-S, pCMV-SARS-S (Sino Biological, Beijing, China) for SARS-S, and pMD.G for VSV-G) by using X-tremeGENE HP transfection reagent (Sigma-Aldrich. The ratio of transfection reagent (μL) to transfected plasmid (μg) was 2:1:2 for packing plasmid pCMVdeltaR8.91, transfer plasmid pLAS2w.RFP-C.Pneo or pLAS2w.FLuc.Ppuro, and surface protein plasmid. The supernatant containing the produced pseudovirus was collected at 96 h after transfection and concentrated by filtering through 100 K Macrosep® centrifugal devices (PALL, Hong Kong, China) at 3000× *g* at 4 °C for 45 min. The titers of pseudoviruses were determined by calculating the infectious dose infecting 50% of tissue culture (TCID_50_/mL). 

### 4.5. Pseudovirus Entry Assay

Eleven cell lines in four groups were inoculated serially ten-fold diluted pseudovirus when the density of cells reached 70% confluence. For the cells inoculated with pseudovirus RFP-Sco-S-eGFP, RFP-SARS-S, or RFP-VSV-G, fresh medium was supplemented after 2 h of inoculation at 37 °C and the status of pseudovirus entry was monitored by observing red fluorescent or green fluorescent cells for three days. For the cells inoculated with pseudovirus FLuc-Sco-S-eGFP, FLuc-SARS-S, or FLuc-VSV-G, the supernatant was discarded and 20 uL of lysis buffer from luciferase assay kit (Promega, Madison, WI, USA) was added. After 20 min of gentle shaking, 100 uL of luciferase reagent was added and FLuc activity (RLU) in cell lysate was measured by Synergy HT microplate reader (Bio-Tek, Winooski, VT, USA). Pseudovirus without the glycoprotein on the surface (△env) was produced by HEK-293T cells co-transfected with packing plasmid and transfer plasmid but without surface protein plasmid and used to inoculate HEK-293T cells as a mock infection control. Each dilution of each pseudovirus inoculated 12 wells and each well was measured three times for average value.

### 4.6. Immunofluorescent Antibody Assay 

At 48 hpi, the pseudovirus-inoculated cells were fixed with the mixture of acetone and methanol (80:20) at room temperature (RT) for 5 min, washed with PBS at RT for 10 min twice and ready for primary antibody staining. To detect the backbone of lentivirus-based pseudovirus, the cells were incubated with 1:100 monoclonal antibody (mAb) to HIV p24 (Millipore, Burlington, MA, USA) as primary antibody at 4 °C overnight, washed with PBS at RT for 10 min twice, incubated with 1:50 fluorescein isothiocyanate (FITC)-conjugated goat anti-mouse IgG (KPL) as secondary antibody at RT for 1 h in dark, washed with PBS at RT for 10 min twice, mounted in DAPI solution (10 mg/L) at RT for 5 min, and covered with cover slip for observation. To detect the GFP fused to S protein of *Scotophilus* bat CoV-512 pseudovirus, the mAb to GFP (Thermo Fisher in 1:200 was used as primary antibody and 1:100 Alexa Fluor^®^ 594 AffiniPure goat anti-mouse IgG (Jackson ImmunoResearch, West Grove, PA, USA) was used as secondary antibody to distinguish the green fluorescence from GFP and red fluorescence from bound secondary antibodies. 

### 4.7. Detection of Cell Receptor 

To detect the cell receptors for CoVs, the polyclonal antibody (pAb) to ACE2, APN, and DPP4 were used in IFA. The cells with 90% confluent density were fixed with the mixture of acetone and methanol (80:20) at RT for 30 min, washed with PBS at RT for 10 min twice, incubated with 5% BSA at RT for 1 h, washed with PBS at RT for 10 min three times, incubated with primary antibody at 4 °C overnight in the dark, washed with PBS at RT for 10 min three times, incubated with FITC-conjugated secondary antibody at RT for 1 h in dark, washed with PBS at RT for 10 min three times, mounted in DAPI solution (10 mg/L) at RT for 5 min, and covered with cover slip for observation. Polyclonal antibodies to three cell receptors were used as primary antibodies: rabbit pAb to human ACE2 (1:200, Abcam, Cambridge, UK), rabbit pAb to human APN (1:100–1:500, Bioss, Boston, MA, USA), goat pAb to human DPP4 (1:66–1:1000, R&D systems, Minneapolis, MN, USA). Secondary antibodies to rabbit and goat primary antibodies were goat anti-rabbit IgG (1:100, KPL, Gaithersburg, MD, USA) and rabbit anti-goat IgG (1:100, KPL). 

### 4.8. Statistical Analysis 

Two-way analysis of variations (ANOVA) with Tukey post hoc test was used to compare RLU values of different cells inoculated the same pseudovirus and multiple *t*-test was used to compare RLU values of each cell to that of the mock infected cell using the GraphPad Prism 6 software package. *p* values of <0.05 were considered as statistically significance. Symbols were assigned as follows: *p* < 0.05, *; *p* < 0.01, **; *p* < 0.001, ***; *p* < 00001, ****.

## 5. Conclusions

Lentivirus-based pseudovirus containing S protein of *Scotophilus* bat CoV-512 or SARS-CoV was generated to determine the susceptibility of 11 different cells for the entry of two CoVs. *Scotophilus* bat CoV-512 pseudovirus had significantly higher entry efficiencies in MDCK, Pabr, and IEC-6 cells and SARS-CoV pseudovirus had significantly higher entry efficiencies in HEK-293, PK15, and MDCK cells. The susceptibility to virus was not completely matched to the distribution to cell receptor APN to PEDV, ACE2 to SARS-CoV, or DPP4 to MERS-CoV. Further characterizations of glycosylation or other factors affecting the interaction between CoV S protein and cell receptor are very important to understand the entry mechanism of CoVs, especially *Scotophilus* bat CoV-512. 

## Figures and Tables

**Table 1:** Summary of cell receptors staining and pseudovirus entry assay.

Cell ^1^	Source	Tissue	APN ^2^	ACE2 ^3^	DPP4 ^4^	FLuc-Sco-S-eGFP ^5^	FLuc-SARS-S ^6^
HEK-293T Caco-2 Vero	Human Human Monkey	Kidney Colon Kidney	>200 Neg.^7^ Neg.	>200 <200 >200	>200 <200 <200	2804 ^efg^ 1715 ^def^ 909 ^fg^	^a^ 4463 ^d^ 2118 ^d^
IEC-6 PK15 MDCK Fcwf-4 Pabr Palu Paki MFK	Rat Pig Dog Cat Fruit Bat Fruit Bat Fruit Bat Bat	Intestine Kidney Kidney Fetus Brain Lung Kidney Kidney	Neg. <200 >200 >200 Neg. Neg. Neg. Neg.	Neg. <200 >200 <200 Neg. Neg. Neg. <200	Neg. <200 Neg. Neg. Neg. Neg. >200 Neg.	3822 ^c^ 2835 ^cd^ 9126 ^a^ 1188 ^efg^ 6708 ^b^ 2225 ^de^ 2270 ^de^ 1683 ^def^	859 ^d^ 24,723 ^b^ 16,024 ^c^ 466 ^d^ 2398 ^d^ 1201 ^d^ 2382 ^d^ 623 ^d^

^1^ Cells in this column are HEK-293T: human (*Homo sapiens*) embryonic kidney epithelial cells; Caco-2: human (*Homo sapiens*) colorectal adenocarcinoma cells; Vero: African green monkey (*Chlorocebus aethiops*) kidney epithelial cells; IEC-6: rat (*Rattus norvegicus*) small intestine epithelial cells; PK15: pig (*Sus scrofa*) kidney epithelial cells; MDCK: Madin Darby dog (*Canis familiaris*) kidney epithelial cells; Fcwf-4: cat (*Felis catus*) whole fetus cells; Pabr: black flying fox (*Pteropus alecto*) brain cells; Palu: black flying fox (*Pteropus alecto*) lung epithelial cells; Paki: black flying fox (*Pteropus alecto*) kidney epithelial cells; MFK: Eastern bent-winged bat (*Miniopterus fuliginosus*) kidney epithelial cells. ^2^ APN: aminopeptidase N. ^3^ ACE2: angiotensin converting enzyme 2. ^4^ DPP4: dipeptidyl peptidase 4. ^5^ FLuc-Sco-S-eGFP: *Scotophilus* bat coronavirus-512 pseudovirus carrying luciferase gene. ^6^ FLuc-SARS-S: severe acute respiratory syndrome coronavirus pseudovirus carrying luciferase gene. ^7^ Neg. means negative immunofluorescent antibody (IFA) assay responses to cell receptors. Average RLU values (n = 12) from cells inoculated with 10^−1^ of pseudoviruses are presented. Statistical significance of differences in RLU values between different cells were tested by two-way ANOVA with Tukey post hoc test and groups with different letters indicate statistical differences (*p* < 0.05).

## Supplementary Materials

The following are available online at https://www.mdpi.com/2076-0817/8/4/259/s1, Figure S1: Cell entry efficiencies of pseudoviruses diluted in 10^−1^ measured by luciferase activity (RLU) at 48 h post infection are shown in different cells.
