# Supplementary material for: Entry of Scotophilus Bat Coronavirus-512 and Severe Acute Respiratory Syndrome Coronavirus in Human and Multiple Animal Cells
Source: Pathogens. 2019 Nov 22;8(4):259. doi: 10.3390/pathogens8040259 (PMC6963420; doi:10.3390/pathogens8040259)
Supplement: Supplementary file 1 [file pathogens-08-00259-s001.pdf]

Supplementary Materials

Figure S1 shows the entry efficiencies of different pseudiviruses in tenth dilution (10^{-1}) on the basis of cell l ines to allow a more direct comparison of virus infectivity in one species. Pseudovirus FLuc-VSV-G had the highest entry efficiencies that other two speudoviruses did in all cells except Palu, where FLuc-Sco-S-eGFP had the highest entry efficiency. No significant differences were observed in Vero, Paki, and MDCK cells infected with FLuc-Sco-S-eGFP and FLuc-SARS-S pseudoviruses, respectively. Pseudovirus FLuc-Sco-S-eGFP had significantly higher entry efficiencies than FLuc-SARS-S did in IEC-6, Pabr, Palu, and Fcwf-4 cells. From evolutionary perspetive, FLuc-Sco-S-eGFP had higher entry efficiencies in bat cells than FLuc-SARS-S did even though FLuc-SARS-S had significantly higher entry efficiency in MFK cells than FLuc-Sco-S-eGFP did.

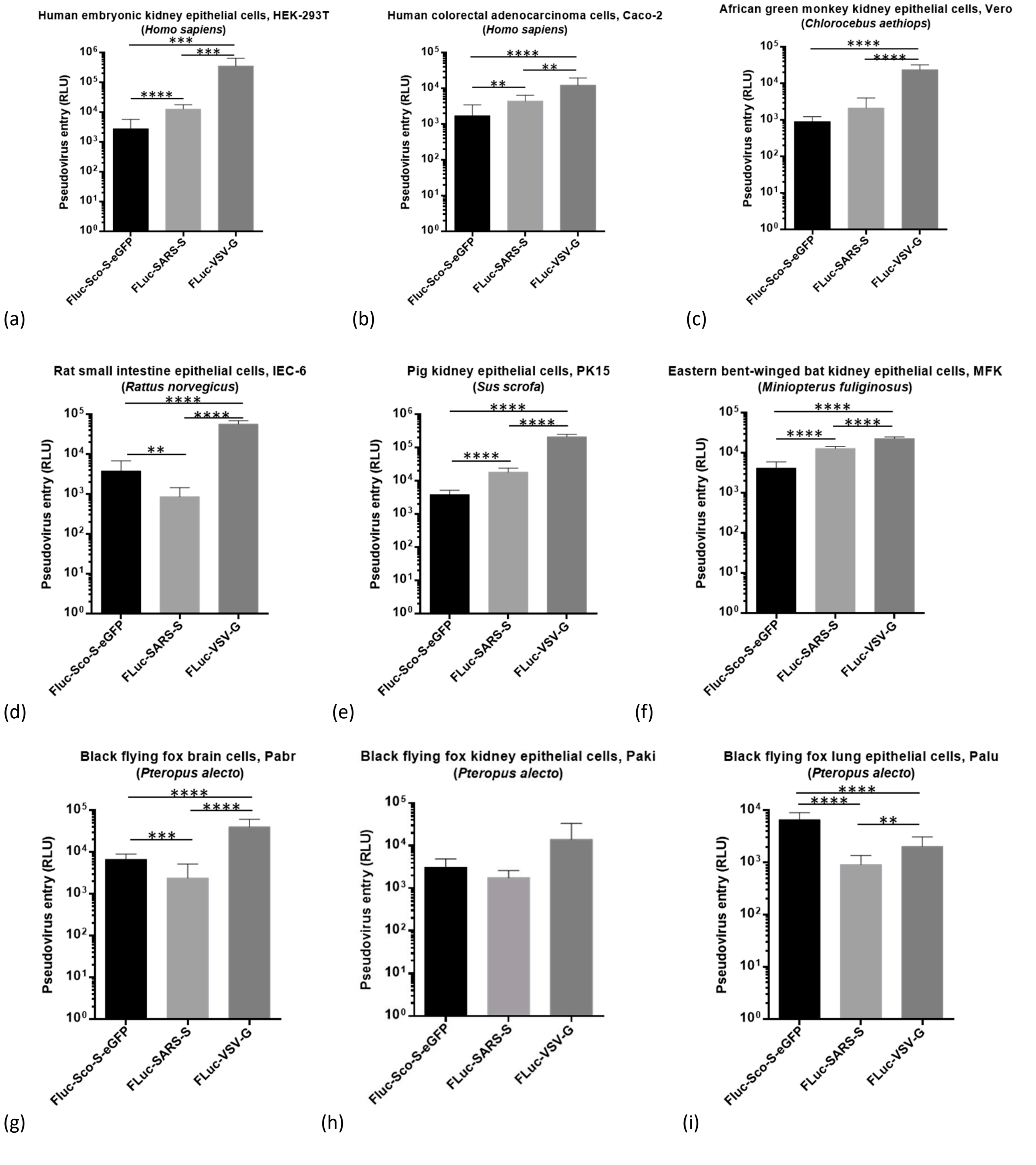

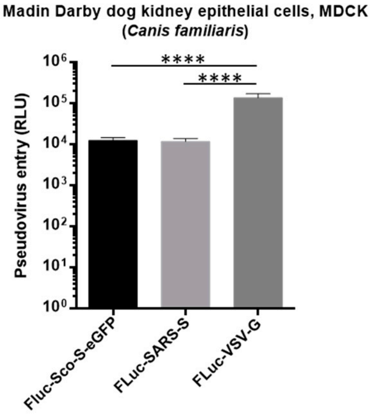

(j)

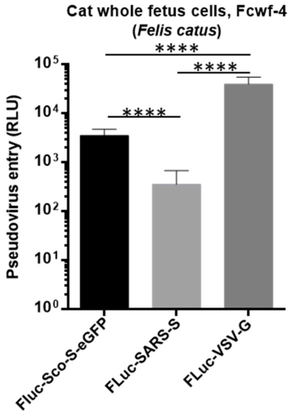

(k)

Figure S1. Cell entry efficiencies of pseudoviruses diluted in 10^{-1} measured by luciferase activity (RLU) at 48 hours post infection are shown in different cells from (a) to (k). Three pseudoviruses are *Scotophilus* bat coronavirus 512 pseudovirus (FLuc-Sco-S-eGFP), severe acute respiratory syndrome coromavirus pseudovirus (FLuc-SARS-S), and vesicular stomatitis virus pseudovirus (FLuc-VSV-G). (a) HEK-293T: human *(Homo sapiens)* embryonic kidney epithelial cells; (b) Caco-2: human *(Homo sapiens)* colorectal adenocarcinoma cells; (c) Vero: African green monkey (*Chlorocebus aethiops*) kidney epithelial cells; (d) IEC-6: rat (*Rattus norvegicus*) small intestine epithelial cells; (e) PK15: pig (*Sus scrofa*) kidney epithelial cells; (f) MDCK: Madin Darby dog (*Canis familiaris*) kidney epithelial cells; (g) Fcwf-4: cat (*Felis catus*) whole fetus cells; (h) Pabr: black flying fox (*Pteropus alecto*) brain cells; (i) Palu: black flying fox (*Pteropus alecto*) lung epithelial cells; (j) Paki: black flying fox (*Pteropus alecto*) kidney epithelial cells; (k) MFK: Eastern bent-winged bat (*Miniopterus fuliginosus*) kidney epithelial cells. Error bars indicate the standard deviation (n = 12). RLU values of different pseudoviruses in each cell were compared by using multiple t tests (*$P < 0.05$; **$P < 0.01$; *** $P < 001$; ****$P < 0.0001$).
